# Supplementary material for: Spread in climate policy scenarios unravelled
Source: Nature. 2023 Dec 13;624(7991):309–16. doi: 10.1038/s41586-023-06738-6 (PMC10719090; doi:10.1038/s41586-023-06738-6)
Supplement: Supplementary file 1 — This file contains Supplementary Sections A1 and A2, additional results. [file 41586_2023_6738_MOESM1_ESM.pdf]

nature portfolio

https://doi.org/10.1038/s41586-023-06738-6

# Supplementary information

# Spread in climate policy scenarios unravelled

In the format provided by the authors and unedited

# Supplementary Information

## SI A. Additional results

### *A.1 Results for 2100*

While the main text focuses on results for 2050, which is of high relevance to policymakers in achieving the Paris climate goals, the same analysis can be done for the second half of the century. Being further away in the future, it is expected that model uncertainties expand, but still, it can provide insights in current robust aspects of energy systems in 2100. Extended Data Figure S3 shows an overview of the results for 2100, analogous to Fig. 3 in the main text.

### *A.2 Absolute versus fractional variables*

Extended Data Figure S4 shows the fractions-based counterpart of Fig. 1 in the main text: variance decomposition results for energy carriers used for electricity generation. No major differences can be observed, making the conclusions we draw in the main text robust under this transformation. In particular, renewable technologies such as solar (yellow) and wind (violet) power, and even the aggregated renewable variable (purple) are all even less climate-dependent and more model dependent than in Fig. 1. This rules out the potential explanation of (e.g.) solar power being model dependent purely because of total absolute energy consumption being model dependent. Interestingly, hydropower now seems rather static across the century with approximately 70% of its variance being determined by model differences, while its variance of absolute values grows from 60% to 80% model-determined in Fig. 1.

Extended Data Figure S5 shows the comparison between variance decomposition results based on the absolute and fractional values, respectively. For most variables, the approach does not change the results significantly, which again strengthens our conclusions in the main text. One exception is primary hydro use, being much less model-dependent in the fractional case (40%) in 2050 than it is in the absolute-value case (65%), which points to that hydropower is sensitive to differences in the total absolute electricity generation. Interestingly, the use of hydropower in electricity generation does not have this effect: even slightly the opposite (71% -> 74%). Nuclear primary energy use and nuclear power both have a decrease in significant model dependency when looking at fractions of the total rather than their absolute values.

Similar to Extended Data Figure S5, we also compare the variance decomposition versions of variable magnitude versus variable fractions of the total for the energy carriers in the end-use sectors. This is shown in Extended Data Figure S6. A number of observations can be made. As mentioned in the main text, electricity use in these sectors become much less model dependent in the fractional case: apparently, the absolute value of electricity use is highly debated, but its relative role is more robust. In industry, hydrogen is 10%-point less model dependent, and in transport, a notable outlier is bioenergy, which moves from 70% of its variance determined by model differences, to only 52%. No notable changes in the “other scenario assumptions” dimension are visible.
